# Micropropagation and Production of Somatic Seeds for Short-Term Storage of the Endangered Species *Eryngium alpinum* L.

**DOI:** 10.3390/plants9040498

**Published:** 2020-04-13

**Authors:** Małgorzata Kikowska, Elwira Sliwinska, Barbara Thiem

**Affiliations:** 1 Department of Pharmaceutical Botany and Plant Biotechnology, University of Medical Sciences in Poznan, 14 Św. Marii Magdaleny St., 61-861 Poznań, Poland; bthiem@ump.edu.pl; 2 Laboratory of Molecular Biology and Cytometry, Department of Agricultural Biotechnology, UTP University of Science and Technology, Prof. S. Kaliskiego Ave. 7, 85-789 Bydgoszcz, Poland; elwira@utp.edu.pl

**Keywords:** alpine eryngo, protected species, clonal propagation, plant growth regulators, somatic seeds production and storage

## Abstract

*Eryngium alpinum* L. is a high-value herb and a source of important compounds that include phenolics, triterpenoid saponins, and essential oils. The present report indicates successful micropropagation of this species. In our study, medium supplemented with BAP 2.0 mg/L, IAA 1.0 mg/L, and GA_3_ 1.0 mg/L was found to be the most suitable for long-term culture and for effective proliferation, irrespective of the passage number. Roots induction, without basal callus formation, was observed when individual microshoots were placed on Murashige & Skoog medium augmented with auxin, and formation was the most advantageous in the presence of NAA alone or when combined with IAA or IBA. The encapsulated propagules were tested for their capability to endure different storage periods under low temperature. Therefore, we developed an efficient method for synseeds production by encapsulation of axillary buds in the sodium alginate matrix, storage for 2, 4, and 6 months, as well as the regeneration process. The maximum regeneration rate of 74% ± 2.72% was observed for axillary buds encapsulated in 4% sodium–alginate complexed with 300 mM calcium chloride after 2 months of storage at low temperature. This is the first report on *E. alpinum* micropropagation and somatic seeds production.

## 1. Introduction

*Eryngium alpinum* L. in the Apiaceae family is protected by Annex II of the Habitats Directive; Appendix I of the Convention on the Conservation of European Wildlife and Natural Habitats; European Habitat Directive Natura 2000; and the national red lists [1,2]. The International Union for the Conservation of Nature (IUCN) classified it as vulnerable [3]. Bad harvesting management, collection for ornamental and commercial use, and the influence of changes in habitats and climate have led to decreases in population, extinction of endangered species, and destruction of natural resources [4]. This alarming situation is raising questions concerning special efforts that should be made to protect plant populations.

Due to the protection status of this taxon, it is not possible to harvest the raw material from natural sites. Conventional propagation by seeds of this species is also difficult because seeds fail to germinate due to a prolonged period of dormancy. Therefore, in vitro cultures can become an alternative source of raw material and allow for the generation of large numbers of propagules from a minimum amount of initial plant material from the natural habitat. The use of in vitro techniques in germplasm conservation is increasing and has been successfully applied to conservation of several rare and endangered species, both for propagation and for long-term storage. Micropropagation as well as somatic seeds are considered to have importance for rapid propagation and ex situ conservation of rare, endemic, and endangered medicinal plants [5]. In vitro cultures bring several advantages: They enable continuous production of uniform biomass from rare and protected plants independent of climatic and environmental conditions. Moreover, plant biomass with good biotechnological parameters may become the material for phytochemical and biological research without the need to deplete natural sites [6].

*E. alpinum* have been examined to a small extent so far. In the organs of intact plants, the presence of several phenolic acids [7,8,9], flavonoids [8,9,10], and essential oil were detected [11]. Chlorogenic acid, rosmarinic acid, and its derivative R-(+)-3′O-β-D-glucopyranosyl rosmarinic acid were found in the root ethanolic extract [7]. 3,4-Dihydroxyphenylacetic, chlorogenic, isochlorogenic, and rosmarinic acids were detected in the methanolic extract of basal leaves [8,9]. Two major flavonoids, kaempferol and quercetin, were found in the aerial part in the study of Crowden et al. [10] whereas isoquercetin was detected in basal leaves in the research of Kikowska et al. [8,9]. The main constituents identified by GC-FID and GC/MS in the essential oils of *E. alpinum* isolated by hydrodistillation of the aerial parts of the plant were caryophyllene oxide and α-bisabolol from oxygenated sesquiterpenes as well as bicyclogermecrene and germacrene D [11].

The aim of the study was to establish an efficient and reproducible micropropagation protocol for alpine eryngo by applying the axillary bud proliferation technique as well as to establish the production of somatic seeds via alginate encapsulated axillary buds and organogenic calli for short-time storage of the endangered species. In the present study, the influence of different plant growth regulators in the agar-solidified media on shoot multiplication and root induction was studied. The effect of the type of a propagule, the bead composition, and the time of storage on regeneration potential of somatic seeds was investigated as well. These methods are especially important for *Eryngium alpinum* L. because it is of high value, its resources are limited based on the availability of plants, and it is difficult to grow. Due to the establishment of in vitro and ex situ collections, multiplication of the desired species may be used to obtain raw material for secondary metabolites production.

To the authors’ best knowledge, there are no other reports on *E. alpinum* micropropagation and somatic seeds production. Alpine eryngo was introduced into in vitro cultures and shoot biomass was cultured in different systems: solid and liquid as well as stationary and agitated, which were previously phytochemically investigated by our team [8,9].

## 2. Results and Discussion

*Eryngium alpinum* L. was introduced into in vitro cultures and the capacity to produce new shoots cultured on the media supplemented with different phytohormones was investigated [8]. Moreover, the suitable systems for *E. alpinum* shoot multiplication were examined [9]. Although the liquid culture systems were reported to be a favorable method for in vitro shoot multiplication, characterized by the possibility of extension for industrial production [12], the development of shoots was relatively high for this species, but shoots were fragile and deformed [9].

The limitation of seed production, endogenous morphological dormancy of seeds, and the low germination rate make generative propagation of this species very inefficient for this taxon [4,13]. For this reason, the small fragments with lateral buds, after disinfection, were the source of explants for the biotechnological experiments.

Growth and the multiplication rate of *E. alpinum* shoots were greatly influenced by the chemical nature of the medium (Table 1).

**Figure 1:**
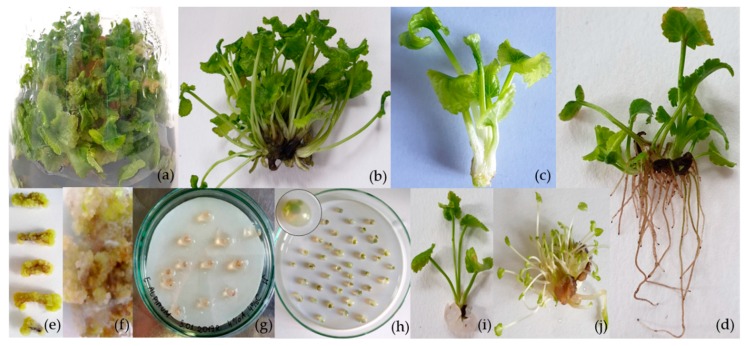
Micropropagation and somatic seeds production of *E. alpinum* L. (**a**) shoots multiplied by axillary buds development; (**b**) multiplied shoots; (**c**) single shoot before root induction; (**d**) plantlets with developed roots; (**e**) leaf-derived callus; (**f**) organogenic callus; (**g**) callus encapsulated; (**h**) axillary buds encapsulated; (**i**) shoot developed from axillary buds encapsulated; (**j**) shoots recovered from organogenic callus encapsulated.

It is known that 6-benzylaminopurine (BAP) is a highly effective regulator of in vitro morphogenesis. It is also well documented that cytokinins, particularly BAP, reduce the apical meristem dominance and stimulate axillary shoot multiplication in numerous species. The need to optimize the concentrations of cytokinins is critical because both the higher and lower concentrations of cytokinins are less effective in new shoot formation [14]. In accordance with our earlier publication [8] aimed at screening for the possibility of in vitro-derived shoots as a good source of phenolic compounds, new shoots developed best not in the presence of BAP alone but with BAP in combination with IAA at half of the BAP concentration. Therefore, in this study, different concentrations of these two regulators of plant growth and development were used in the systems in which the cytokinin concentration was the same or twice as high as that of auxin. Gibberellic acid (GA_3_), the most common gibberellin applied, was used for inducement of shoot elongation. In our study, the medium supplemented with BAP 2.0 mg/L, IAA 1.0 mg/L, and GA_3_ 1.0 mg/L was found to be the most suitable for long-term culture of *E. alpinum*, followed by BAP 1.0 mg/L, IAA 0.5 mg/L, and GA_3_ 1.0 mg/L for effective multiplication, irrespective of the passage number (Table 1). Shoots grew vigorously and did not show any signs of vitrification or callusing at the base, irrespective of the plant growth regulators (PGRs) used (Figure 1). The higher multiplication ratio for this species was 18.09 ± 1.43–25.12 ± 2.12 depending on subculture, but during several passages proved to be the most efficient (Table 1). In the case of *E. planum*, the highest mean number of shoots developed from axillary buds was 15.58 ± 0.54 − 17.10 ± 0.60 shoots depending on the Murashige & Skoog culture media: MS + BAP 1.0 mg/L + IAA 1.0 mg/L or MS + BAP 1.0 mg/L + IAA 0.1 mg/L [15]. For *E. campestre*, more shoots (13.30 ± 3.73) were obtained when cultured on the same media [16]. The efficiency of shoot multiplication for *E. maritimum* varied between 1.2 ± 0.20 and 4.4 ± 0.24 shoots per explant on the different media variants [17].

It was observed in our previous studies [8,9] that, contrary to other related *Eryngium* species, *E. planum* [15], *E. campestre* [16], and *E. maritimum* [17], roots of alpine eryngo did not spontaneously occur on the media that were intended for shoot multiplication and which were characterized by the higher cytokinin concentration or the equal concentration of cytokinin and auxin. Application of BAP, even in the optimum concentrations, can inhibit subsequent root initiation [18]. In addition, the control medium, without phytohormones, was of little benefit for root induction and development, and the addition of auxins significantly affected rhizogenesis. In vitro-multiplied shoots induced roots in a way that depended on the type of auxin and their combination (Figure 1, Table 2).

It can be concluded from the results presented in Table 2 that the addition of individual auxins to the rooting medium resulted in a different explant response. Supplementation of MS medium with picloram did not affect the appearance of roots, and when considering that roots were formed on the auxin-free media, it can be concluded that picloram inhibited their formation. The best type of auxin added to the medium was NAA, which induced on average 27 roots per shoot. Roots were characterized by similar morphology as those observed for *E. planum*, *E. campestre*, and *E. maritimum* in the presence of NAA: relatively thick and dark, initially short. However, unlike the compared species, callus did not form at the base of shoots or on the induced roots of *E. alpinum*. Profusely branched roots observed for micropropagated plantlets of *E. planum*, *E. campestre*, and *E. maritimum* as a result of IBA supplementation were not observed for *E. alpinum* [15,16,17]. Another effective auxin was 2,4-D and under its influence an average of 15 roots formed per cultured explant. Combinations of auxins were shown to be highly beneficial in the process of shoot rooting. It was observed that combinations inducing the highest number of roots (almost 29 and 35 per shoot) contained the addition of NAA. In previous studies on micropropagation of other *Eryngium* species, no auxin combination was used in the rooting stage of shoots.

The survival rate for clonally propagated *E. alpinum* was relatively low compared to other *Eryngium* species investigated by our team [15,16,17]. Sixty percent (36 plantlets) of rooted plants survived after being transplanted in the pots and 39% (14 plantlets) in the soil. In vitro micropropagated plants were morphologically uniform, vigorous, and had well-developed roots. No detectable differences in growth characteristics were observed between clonal propagated plants and intact plants.

In the present work, a combination of Dic 1.0 mg/L and TDZ 1.0 mg/L supported *E. alpinum* callus induction. With the aim of inducing shoot bud organogenesis, the callus obtained was placed on MS medium supplemented with BAP 1.0 mg/L, IAA 1.0 mg/L, and GA_3_ 1.0 mg/L. Finally, organogenic calli provided valuable material for short-term storage of somatic seeds.

Formation of beads with appropriate stability and hardness is of key importance for producing somatic seeds: very hard beads limit the regeneration ability, while soft beads dissolve without protecting the encapsulated propagules. The type of explant, the concentrations of sodium alginate (SA) and calcium chloride (CC), and complexation duration were studied for *E. alpinum* synseeds in this experiment (Table 3).

Out of the three different concentrations of SA (2%, 3%, 4%) and the three concentrations of CC (100, 200, 300 mM) evaluated to develop the encapsulation matrix, 4% SA and 100 mM CC were the most appropriate for beads production and somatic seeds storage. The lower concentrations resulted in a weak structure, non-round shape of beads in nature, and poorly-coated propagules, which resulted in browning. Encapsulation with 4% SA and 100 mM CC was found to be very effective in maintaining the viability of the propagules and regeneration potential of plantlets.

Axillary buds of in vitro-raised plants as well as ogranogenic calli were used as the propagules for encapsulation (Figure 1, Table 4). After storage, regardless of the number of weeks, encapsulated axillary buds showed the earliest response after 14 days compared to clumps of calli, which initially regenerated shoots after 24 days. Generally, axillary buds were more effective explants in regenerating shoots and then roots than were calli. The maximum regeneration rate of 74% ± 2.72% was observed for axillary buds encapsulated in 4% sodium–alginate with 30 min complexation with 300 mM calcium chloride after 2 months of storage at low temperature. Somatic seeds prepared from calli coated in 4% SA and 300 mM CC exhibited the lowest regeneration rate (15% ± 0.71%) after 6 months of low-temperature storage. The regeneration rate, regardless of explants used, subsequently decreased with the increased duration (Table 4). In the study, a rupture of the encapsulation matrix during storage was not observed. Instead, in contrast to axillary buds, the callus fragments turned yellowish with the increase in storage duration.

Somatic seeds production of a wide range of important endangered and protected species is considered an effective way to support their conservation [19,20,21].

The confirmation of genome size stability is of importance during in vitro plants propagation. In our study, the 2C DNA content of E. alpinum established in different plant material, namely leaves from shoots developed from axillary buds as well as regenerated from calli, encapsulated propagules, and in calli, was similar and ranged from 2.32 to 2.43 pg/2C (Table 5). The results concerning genome size stability were notably consistent with our previous studies on other Eryngium species [15,16,17].

## 3. Materials and Methods

### 3.1. The Plant Material

Young individuals of *Eryngium alpinum* L. were collected from the Botanical Garden of Adam Mickiewicz University in Poznań, in September 2017. The voucher specimen was deposited in the Department of Pharmaceutical Botany and Plant Biotechnology Poznań University of Medical Sciences under the number H-AP-2017-102. Primary explants, shoot fragments with lateral buds, were surface disinfected according to the procedure adopted by [8]. Briefly, explants were disinfected with 50% (*v*/*v*) commercial bleach solution with 3.3% active calcium hypochlorite and 2–3 drops of Tween 80 for 5 min.

### 3.2. The Culture Media and Conditions

Aseptic explants were placed in Erlenmeyer flasks with 50 mL of solidified MS medium [22] with PGRs, benzylaminopurine (BAP; Sigma-Aldrich, Saint Louis, USA), indolile-3-acetic acid (IAA; Sigma-Aldrich, Saint Louis, MO, USA), and gibberellic acid (GA_3_; Sigma-Aldrich, Saint Louis, MO, USA), at the concentration of 1.0 mg/L [8]. The media were autoclaved at 121 °C, at a pressure of 0.1 MPa for 20 min. The cultures were grown under artificial light at 55 µmol/m^2^s (16 h light/8 h dark photoperiod) and at temperature of 21 °C ± 2 °C.

### 3.3. Micropropagation: Shoot Multiplication

Shoots were multiplied via the axillary branching method [23] on MS medium solidified with 7.6 g/L agar and enriched with BAP (2.0 mg/L, 1.0 mg/L or 0.5 mg/L), IAA (1.0 mg/L or 0.5 mg/L), and GA_3_ (2.0 mg/L, 1.0 mg/L or 0.5 mg/L) for 24 passages. The 250 mL Erlenmeyer flasks with 50 mL of the medium were used. After 40 days of culture, the percentage of explant-regenerated shoots, the number of new shoots per explant and their length were measured. The experiments were repeated three times for 30 explants at different time intervals.

### 3.4. Micropropagation: Root Induction

In vitro-multiplied shoots of c.a. 2–3 cm (from 12th to 18th subculture) were transferred to MS solidified medium supplemented with a single auxin: indolile-3-acetic acid (IAA), indolile-3-butyric acid (IBA, Sigma-Aldrich, Saint Louis, MO, USA), 1-naphthaleneacetic acid (NAA, Sigma-Aldrich, Saint Louis, MO, USA), 2,4-dichlorophenoxyacetic acid (2,4-D, Sigma-Aldrich, Saint Louis, MO, USA), 4-amino-3,5,6-trichloro-2-pyridinecarboxylic acid (picloram, Pic, Sigma-Aldrich, Saint Louis, MO, USA), 3,6-dichloro-2-metoxybenzoic acid (dicamba, Dic, Sigma-Aldrich, Saint Louis, MO, USA), or their combination IAA + IBA, IAA + NAA, IBA + NAA, each at the concentration of 1.0 mg/L. The glass tubes with 30 mL of the medium were used. The number of roots per shoot and their length were recorded after 40 days. The experiments were repeated three times for 10 explants at different time intervals.

Successfully rooted plantlets were removed from the rooting medium, washed thoroughly to remove adhering agar, and transferred to micro plastic pots of 5 cm height and 6 cm diameter containing a mixture of soil, vermiculite, and sand (1:1:1 *v*/*v*/*v*) and covered with translucent plastic cubs to ensure high humidity. The pots with in vitro propagated plantlets (60 individuals) were placed at room temperature (23 ± 2 °C) under normal day length conditions. The survival rate was recorded after 62 days and then the plantlets were transferred to the field (36 individuals).

### 3.5. Organogenic Callus Induction and Proliferation

Leaf explants of multiplied shoots were placed in 250 mL flasks containing 50 mL of the solidified MS basal medium with 3,6-dichloro-2-metoxybenzoic acid (dicamba, Dic 1.0 mg/L) and thidiazuron (TDZ 1.0 mg/L, Sigma-Aldrich, Saint Louis, MO, USA). Callus clumps observed after three weeks of culture on MS medium with Dic and TDZ were divided into clumps of about the same size (1 cm^2^) and transferred onto the regeneration MS media with the selected PGRs: BAP (1.0 mg/L), IAA (1.0 mg/L), and GA_3_ (1.0 mg/L). Organogenic callus cultures were grown under the same light and temperature conditions as the shoot cultures developed from lateral buds.

### 3.6. Synthetic Seed Production and Storage

Axillary buds and organogenic calli of a 0.3–0.4 cm diameter were excised from in vitro cultures. For encapsulation, explants were plunged into the solution of sodium alginate and then into calcium chloride (CaCl_2_ × 2H_2_O) for complexation. Somatic seeds were washed with sterile distilled water. The encapsulated propagules were placed in 90 cm diameter Petri dishes at 4 °C in darkness and after the storage time they were transferred to the growth chamber for recovery. MS medium supplemented with BAP 2.0 mg/L, IAA 1.0 mg/L, and GA_3_ 1.0 mg/L was used for plant regeneration of somatic seeds. The types of plant growth regulators and their concentrations were optimized through a separate experiment: shoot multiplication and callus proliferation.

In the first experiment, different concentrations of sodium alginate solution, 2% (*w*/*v*), 3% (*w*/*v*) or 4% (*w*/*v*), and calcium chloride, 100 mM, 200 mM, and 300 mM, were tested. Different time periods (20, 30, or 40 min) for Na^+^/Ca^2+^ ion exchange in the calcium chloride solution were applied (no statistical differences were noted for the results, therefore they were not included in the article). In the second experiment, different storage times of somatic seeds (2, 4, 6 months) placed in 90 cm diameter Petri dishes at 4 °C in darkness were tested. Each experiment was repeated three times for c.a. 40 explants.

### 3.7. Genome Size Estimation

Leaves of in vitro-multiplied shoots, shoots regenerated from calli, callus biomass, and leaves of shoots regenerated from the alginate-encapsulated propagules after storage were used for flow cytometric estimation of nuclear DNA content. A nuclei isolation buffer [24] supplemented with 1% (*v*/*v*) polyvinylpyrrolidone (PVP-10), propidium iodide (PI; 50 µg/cm^3^), and ribonuclease A (50 µg/cm^3^) was applied for sample preparation. *Petunia hybrida* P × Pc6 (2.85 pg/2C; [25]) was used as an internal standard. For each sample, 5000–8000 nuclei were analyzed using a CyFlow SL Green flow cytometer (Partec GmbH, Münster, Germany) equipped with a high-grade solid-state laser with green light emission at 532 nm, a long-pass filter RG 590 E, DM 560 A, as well as with side (SSC) and forward (FSC) scatters, using linear amplification. FloMax software (Partec GmbH, Münster, Germany) was applied for histogram evaluation. The analyses were replicated at least three times for each plant material. The coefficient of variation (CV) of G0/G1 peak of *E. alpinum* ranged from 3.2% to 4.6%. The nuclear DNA content was calculated using the linear relationship between the ratio of the 2C peak positions *E. alpinum*/*P. hybrida* on a histogram of fluorescence intensities.

### 3.8. The Statistical Analysis

The obtained data were analyzed using a one-way analysis of variance (ANOVA) and the statistical significance was determined by Duncan’s POST-HOC test (*p* value of 0.05). All the analyses were conducted using STATISCICA v. 13 (StatSoft Polska, Inc. 2015, Kraków, Poland).

## 4. Conclusions

The present report details the efficient protocol for *Eryngium alpinum* L. micropropagation from axillary bud development with the confirmed uniformity of genome size. Micropropagation and synseeds are encouraging methods for germplasm conservation of valuable and uniform raw material of endangered and protected species. Our study indicated that somatic seeds, artificially encapsulated with 4% sodium alginate and 300 mM calcium chloride axillary buds, could be stored at 4 °C for 6 months without loss of viability and with high regeneration ability.

## Figures and Tables

**Table 1:** The effects of tested plant growth regulators on *Eryngium alpinum* L. growth parameters.

MS Medium Supplementation			Growth Parameters		
Plant Growth Regulators					
Cytokinin (mg/L)	Auxin (mg/L)	Gibberellin (mg/L)	Induction (%)	Shoot No./Explant ± SE	Shoot Length (CM) ± SE
			**Sixth Subculture**		
BAP [2.0]	IAA [1.0]	GA_3_ [2.0]	100	4.70 ± 0.08 ^g^	3.87 ± 0.22 ^b^
BAP [2.0]	IAA [1.0]	GA_3_ [1.0]	100	24.10 ± 1.35 ^a^	2.12 ± 0.05 ^e^
BAP [1.0]	IAA [0.5]	GA_3_ [1.0]	100	13.70 ± 0.70 ^dc^	2.05 ± 0.15 ^e^
BAP [0.5]	IAA [0.5]	GA_3_ [0.5]	80	7.50 ± 0.35 ^ef^	3.43 ± 0.16 ^c^
			**Twelfth Subculture**		
BAP [2.0]	IAA [1.0]	GA_3_ [2.0]	100	3.50 ± 0.28 ^g^	4.57 ± 0.13 ^a^
BAP [2.0]	IAA [1.0]	GA_3_ [1.0]	100	18.90 ± 0.80 ^b^	2.23 ± 0.09 ^e^
BAP [1.0]	IAA [0.5]	GA_3_ [1.0]	100	15.20 ± 0.63 ^c^	2.31 ± 0.04 ^e^
BAP [0.5]	IAA [0.5]	GA_3_ [0.5]	100	8.10 ± 0.48 ^e^	3.80 ± 0.08 ^b^
			**Eighteenth Subculture**		
BAP [2.0]	IAA [1.0]	GA_3_ [2.0]	100	5.40 ± 0.58^fg^	3.43 ± 0.01^c^
BAP [2.0]	IAA [1.0]	GA_3_ [1.0]	100	25.10 ± 1.38 ^a^	2.16 ± 0.05 ^e^
BAP [1.0]	IAA [0.5]	GA_3_ [1.0]	100	15.10 ± 1.19 ^c^	2.09 ± 0.03 ^e^
BAP [0.5]	IAA [0.5]	GA_3_ [0.5]	80	5.60 ± 0.16 ^fg^	4.08 ± 0.09 ^b^
			**Twenty Fourth Subculture**		
BAP [2.0]	IAA [1.0]	GA_3_ [2.0]	80	5.20 ± 0.29 ^fg^	4.76 ± 0.08 ^a^
BAP [2.0]	IAA [1.0]	GA_3_ [1.0]	90	20.10 ± 0.74 ^b^	2.08 ± 0.04 ^e^
BAP [1.0]	IAA [0.5]	GA_3_ [1.0]	100	12.50 ± 1.45 ^d^	3.01 ± 0.03 ^d^
BAP [0.5]	IAA [0.5]	GA_3_ [0.5]	100	5.60 ± 0.31 ^fg^	3.42 ± 0.04 ^c^

**BAP**: 6-benzylamninopurine, **IAA**: indolile-3-acetic acid, **GA_3_**: gibberellic acid. Mean values within a column with the same letter are not significantly different at *p* = 0.05 (Duncan’s multiple range test).

**Table 2:** The effect of various types of auxins supplementing Murashige & Skoog media on root induction and development of *Eryngium alpinum* L. shoots after 40 days of culture.

Auxin(s) (1.0 mg/L)	Induction (%)	Mean No. of Roots ± SE	Mean Root Length ± SE
-	50	1.4 ± 0.64 ^e^	1.96 ± 0.17 ^cd^
IAA	100	2.9 ± 0.86 ^de^	2.36 ± 0.12 ^a^
IBA	100	7.5 ± 2.23 ^d^	1.77 ± 0.07 ^cd^
NAA	100	27.4 ± 1.35 ^b^	0.61 ± 0.04 ^b^
2,4-D	100	15.00 ± 2.69 ^c^	1.79 ± 0.06 ^cd^
Dic	100	3.00 ± 1.53 ^de^	1.68 ± 0.12 ^d^
Pic	0	0.00 ± 0.00 ^e^	0.00 ± 0.00 ^e^
IAA + IBA	100	20.2 ± 2.28 ^c^	2.09 ± 0.05 ^a^
IAA + NAA	100	34.5 ± 2.04 ^a^	0.80 ± 0.04 ^b^
IBA + NAA	100	28.9 ± 2.79 ^b^	0.90 ± 0.04 ^b^

**2,4-D**: 2,4-dichlorophenoxyacetic acid, **Dic**: (Dicamba) 3,6-dichloro-2-metoxybenzoic acid, **IAA**: indolile-3-acetic acid, **IBA**: indolile-3-butyric acid, **NAA**: 1-naphthaleneacetic acid, **Pic**: (Picloram) 4-amino-3,5,6-trichloro-2-pyridinecarboxylic acid. Mean values within a column with the same letter are not significantly different at *p* = 0.05 (Duncan’s multiple range test).

**Table 3:** The influence of the type of a propagule, the bead composition, as well as storage duration (4 °C) on somatic seeds formation of *Eryngium alpinum* L.

Propagules	Sodium Alginate	Calcium Chloride	Bead Characteristic
Axillary buds	2%	100 mM	Too soft to handle
Axillary buds	2%	200 mM	Too soft to handle
Axillary buds	2%	300 mM	Too soft to handle
Axillary buds	3%	100 mM	Formed tails
Axillary buds	3%	200 mM	Formed tails
Axillary buds	3%	300 mM	Too squashy
Axillary buds	4%	100 mM	Deformed and isodiametric beads
Axillary buds	4%	200 mM	Deformed and isodiametric beads
Axillary buds	4%	300 mM	Isodiametric beads
Organogenic callus	2%	100 mM	Too soft to handle
Organogenic callus	2%	200 mM	Too soft to handle
Organogenic callus	2%	300 mM	Too soft to handle
Organogenic callus	3%	100 mM	Formed tails
Organogenic callus	3%	200 mM	Formed tails
Organogenic callus	3%	300 mM	Too squashy
Organogenic callus	4%	100 mM	Deformed and isodiametric beads
Organogenic callus	4%	200 mM	Deformed and isodiametric beads
Organogenic callus	4%	300 mM	Isodiametric beads

**Table 4:** The influence of the type of explant and storage duration (4 °C) on recovery of the encapsulated propagules of *Eryngium alpinum* L.

Propagules	Sodium Alginate	Calcium Chloride	Storage Duration	Survival Percentage	Recovery Percentage (±SE)
Axillary buds	4%	300 mM	0 months	100	100 ± 0.00 ^a^
Axillary buds	4%	300 mM	2 months	100	74 ± 2.08 ^b^
Axillary buds	4%	300 mM	4 months	90	56 ± 1.83 ^c^
Axillary buds	4%	300 mM	6 months	60	42 ± 1.04 ^d^
Organogenic callus	4%	300 mM	0 months	80	46 ± 0.90 ^e^
Organogenic callus	4%	300 mM	2 months	60	32 ± 0.97 ^f^
Organogenic callus	4%	300 mM	4 months	40	26 ± 0.76 ^g^
Organogenic callus	4%	300 mM	6 months	20	15 ± 0.44 ^h^

Mean values within a column with the same letter are not significantly different at *p* = 0.05 (Duncan’s multiple range test).

**Table 5:** The nuclear DNA content in leaves of micropropagated plantlets and calli of *Eryngium alpinum* L.

Plant Material	DNA Content (PG/2C) ± SE
Leaf of shoot culture at passage 6	2.35 ± 0.01 ^ns^
Leaf of shoot culture at passage 12	2.35 ± 0.01
Leaf of shoot culture at passage 18	2.35 ± 0.00
Leaf of shoot culture at passage 24	2.32 ± 0.01
Callus	2.36 ± 0.01
Leaf of callus-derived shoots	2.43 ± 0.04
Leaf of encapsulated callus-derived shoots	2.35 ± 0.01
Leaf of encapsulated bud-derived shoots	2.34 ± 0.01

ns—no significant differences at *p* = 0.05 (Duncan’s multiple range test).
